# Thresholds for Oxford Knee Score after total knee replacement surgery: a novel approach to post-operative evaluation

**DOI:** 10.1186/s13018-017-0592-1

**Published:** 2017-06-12

**Authors:** Christian Lund Petersen, Jonas Bruun Kjærsgaard, Nicolai Kjærgaard, Michael Ulrich Jensen, Mogens Berg Laursen

**Affiliations:** 1 Department of Orthopaedic Surgery, Aalborg University Hospital, Hobrovej 18-22, 9000 Aalborg, Denmark; 2 School of Medicine and Health, Aalborg University, Niels Jernes Vej 12 A5, 9220 Aalborg Ø, Denmark; 3 Helgolandsgade 14, 2th, 9000 Aalborg, Denmark

**Keywords:** PROM, Oxford Knee Score, OKS, Knee arthroplasty, Knee, Total knee replacement

## Abstract

**Background:**

In a prospective cohort study, we wanted to detect thresholds distinguishing between patients with a satisfactory and an unsatisfactory outcome after total knee replacement (TKR) based on Patient-Reported Outcome Measures (PROMs), namely the Oxford Knee Score (OKS), using patient satisfaction and patient-perceived function as global transition items.

**Methods:**

Seventy-three TKR patients completed the OKS questionnaire before surgery and were invited to complete the same questionnaire again 6 (4 to 9) months after surgery. Correlations between outcome measures and anchors were calculated using Pearson’s correlation coefficient. Thresholds were established by receiver operating characteristics (ROC) analysis, using multiple anchor-based approaches.

**Results:**

Patients showed a mean increase of 16.5 (SD 9.5) in OKS following TKR. Significant positive correlations were found between outcome measures and anchors. Six different thresholds were determined for outcome measures coupled with satisfaction, patient-perceived function and a combination thereof using a cut-off of 50 and 70.

**Conclusions:**

This study has established a set of clinically meaningful thresholds for Oxford Knee scores that may help to detect TKR patients who might be in need of post-operative evaluation.

## Background

Traditionally, when evaluating the quality of total knee replacements (TKR), indicators such as survival of the prosthesis and revision rates have been used as standard measurements [1]. However, in recent years, Patient-Reported Outcome Measures (PROMs) have gained increased attention when evaluating outcomes of TKR [2, 3]. Joint-specific PROMs allow the assessment of the outcome from the perspective of the patient, including the level of pain and function of the specific joint.

One such PROM was devised in 1998. At the time of introduction of the Oxford Knee Score (OKS), the scoring system was developed as a measure of post-operative outcome for TKR [4]. Used in cohort studies and collected in national registries, such as in England and Wales, Sweden and New Zealand [5, 6], it has since been coupled to other patient-reported measures allowing a more comprehensive assessment of TKR outcomes [2, 6]. This simplifies the interpretation of the quantitative score into qualitatively meaningful information [7].

Thresholds can be established for OKS values above which patients are satisfied with surgery or have experienced improvement of function after surgery. Multiple methodological approaches to calculating such thresholds exist. One approach is called the minimal clinically important difference (MCID), which is defined as “the smallest change that is important to patients” [8]. Another approach is calculating a threshold of the post-operative OKS value, providing another perspective of patient-perceived outcome.

These approaches require the use of global transition items as anchors. Previous studies have used patient satisfaction with surgery and perceived change in function of the specific joint as anchors [2, 6–9].

Previous studies have identified OKS thresholds to aid the clinician in presenting the expected outcome of surgery in a meaningful way to the patient [6]. However, the thresholds may have other possible applications. As the use of Oxford scores provides a means of comparing preoperative and post-operative health status, they may be used as a tool in the process of determining which patients are in need of further post-operative treatment.

In Danish hospitals, there is no standardised method for identifying TKR patients in need of further post-operative treatment. Current methods range from yearly outpatient visits in the surgeon’s office to nurse-performed structural phone interviews using a modified version of the American Knee Society Score (AKSS) with defined triggering responses [10]. This is very time consuming, and the proportion of patients in need of re-evaluation is relatively small and hence does not fully satisfy the time and resources spent.

A screening procedure using OKS as part of a web-based questionnaire is planned to be used as a tool to select patients for outpatient evaluation in the North Denmark Region. Thus, this paper is a pilot study intended to create an initial algorithm intended to choose which patients should be called in for outpatient evaluation 1 year after surgery.

Based on the above considerations, we hypothesise that it is possible to identify clinically meaningful thresholds for OKS determining which patients are in need of post-operative evaluation.

## Methods

Data were obtained from a clinical quality database (“Jointbase”) at the Department of Orthopaedic Surgery, Aalborg University Hospital. The purpose of this database is to prospectively monitor the results of hip and knee arthroplasty surgery. This is assessed through a questionnaire using a condition-specific instrument (OKS), a generic instrument (EQ-5D-3L) and pain measurements.

All patients who completed the questionnaire prior to their surgery and underwent TKR (*n* = 73) at Aalborg University Hospital in the period between May 1 and October 31, 2014, were included in the study. Patients were invited to a follow-up investigation during February and March 2015. At this visit, the preoperative questionnaire was repeated in order to identify changes in the aforementioned scores. Additionally, patients completed a post-operative form, which included two global transition items.

### Outcome measures and global transition items

Joint-specific PROMs were collected using the Danish translation of OKS [11]. The OKS is a 12-item questionnaire assessing pain and function in the patient’s knee during the last 4 weeks.

Current overall satisfaction with the outcome of surgery was evaluated by a bipolar visual analogue scale (VAS) from 0 (very unsatisfied) to 100 (very satisfied). Present patient-perceived function in the knee compared with before the surgery was assessed by a bipolar VAS from 0 (much worse) to 100 (much better).

### Statistical analysis

Descriptive statistics were performed for attenders and non-attenders. The attenders were compared to the non-attenders by chi-squared tests for categorical variables and two-sample *t* tests for continuous variables. To support the conclusions of the two-sample *t* tests, permutation tests were conducted.

Correlations between satisfaction with surgery and post-operative OKS, change in OKS was calculated using Pearson’s correlation coefficient. Correlations with patient-perceived function were calculated in the same manner.

Using a sensitivity- and specificity-based approach, [8] thresholds were calculated for change in OKS (ΔOKS) and absolute post-operative OKS by using two global transition items for constructing three anchors: patient satisfaction, patient-perceived function and a combination of the two former by using the most conservative value, i.e. the lowest value hereof.

Cut-off points of 50 and 70 for patient satisfaction with surgery were chosen, and thus define a binary outcome: patients with satisfaction values below the cut-off should be invited for out-patient evaluation and patients with values above the cut-off should not. Likewise, cut-off points of 50 and 70 for patient-perceived function in the knee in question were used. Finally, thresholds were calculated by defining the cut-off as 50 or 70 for the combined anchor. In other words, patients who scored below the cut-off in either one of the two global transition items were identified as patients who should be invited for out-patient evaluation. Thus, we do not seek to identify patients that are, e.g. 100% satisfied, but merely discriminate between the two groups of patients based on a score of either below or above the cut-off, i.e. 50 or 70.

Coupling the anchors to the outcome measures (ΔOKS, OKS), sensitivity and specificity for different threshold values were assessed by receiver operating characteristics (ROC) curves plotting sensitivity against specificity.

Thresholds were established for each outcome measure by identifying the point on the relevant ROC curve closest to the upper left corner, as this represents the most efficient threshold value with regard to specificity and sensitivity [7].

Furthermore, the area under the curve (AUC) was calculated. The AUC represents the probability that the outcome measure threshold value correctly discriminates between patients who do and do not reach the cut-off. An AUC between 0.7 and 0.8 is considered acceptable, and an AUC between 0.8 and 0.9 is considered excellent [8].

Statistical analysis was performed using R version 3.1.3 [12].

## Results

### Study population characteristics

A total number of 73 TKR patients were included in the study of which 57 patients (78%) attended the post-operative follow-up. Patients were seen at an average of 6.05 (SD 1.62) months after surgery.

Attenders and non-attenders were analysed for differences between groups. Analyses regarding gender, age, preoperative OKS and body mass index (BMI) revealed no statistically significant differences. Descriptive statistics and *p* values are shown in Table 1.

**Table 1:** Comparison of preoperative OKS, age, BMI and gender of attenders and non-attenders. *P* values from two-sample *t* test unless otherwise stated

	TKR patients		
	Attenders	Non-attenders	
	(*n* = 57, 78.1%)	(*n* = 16, 21.9%)	*p* value
Pre-operative OKS, mean (SD)	20.3 (6.9)	18.4 (7.6)	0.36
Age (years), mean (SD)	65.6 (11.5)	66.1 (12.0)	0.88
BMI, preoperative, mean (SD)	30.5 (5.6)	30.6 (5.0)	0.95
Gender (*n*, %)			1^a^
Male	20 (35.1)	6 (37.5)	
Female	37 (64.9)	10 (62.5)	

*SD* standard deviation

^a^Chi-squared test

### Post-operative improvements and correlation with global transition items

On average, patients showed an increase of 16.5 (SD 8.5) in OKS, demonstrating an improvement in knee function after TKR (*p* < 0.01). The mean OKS before surgery was 20.3 (SD 6.9) and 36.8 (SD 6.8) after surgery.

Significant correlations were found between global transition items (patient satisfaction or patient-perceived function) and outcomes (post-operative OKS, change in OKS) as assessed by simple linear regression and derived by Pearson’s correlation coefficient.

Positive correlations were found between satisfaction and post-operative OKS (*r* = 0.56 (CI 0.35; 0.71)) and between satisfaction and change in OKS (*r* = 0.42 (CI 0.17; 0.61)).

The same pattern is seen for correlations with function. Post-operative OKS (*r* = 0.56 (CI 0.35; 0.71)) and change in OKS (*r* = 0.42 (CI 0.17;0.61)) are positively correlated to function.

### Anchors and cut-off values

Using a cut-off of 50 for satisfaction, the study identified 91.2% (52/57) TKR patients as being satisfied. 84.2% (48/57) were identified as satisfied when using a cut-off of 70.

Patient-perceived function cut-offs of 50 and 70 revealed function gain in 91.2% (52/57) and 78.9% (45/57) of patients, respectively.

The combined anchor cut-offs identified 86.0% (49/57) of patients as above 50 and 73.7% (42/57) of patients as above 70.

### Thresholds for outcomes after surgery

Thresholds for various outcome measures identified by ROC-curves at cut-off values of 50 and 70 for satisfaction and patient-perceived function are presented in Tables 2 and 3.

**Table 2:** Thresholds, percentage of patients who will be called with the given threshold, specificity, sensitivity and area under curve (AUC) for OKS and ΔOKS anchored to patient-perceived satisfaction, function and either satisfaction or function with a cut-off of 50. True positives is the amount of patients who should be called according to the cut-off value

	Cut-off value 50				
Anchor	Threshold	Called (%)	Specificity	Sensitivity	AUC
Satisfaction (*n* = 5)					
Post-operative OKS	34.5	31.6	0.75	1.00	0.88
ΔOKS	9.5	22.8	0.83	0.80	0.86
Function (*n* = 5)					
Post-operative OKS	32.5	24.6	0.81	0.80	0.85
ΔOKS	9.5	22.8	0.83	0.80	0.89
Satisfaction or function (*n* = 9)					
Post-operative OKS	34.5	31.6	0.78	0.88	0.87
ΔOKS	9.5	22.8	0.86	0.75	0.88

*n* number of patients with anchor values below the cut-off

**Table 3:** Thresholds, percentage of patients who will be called with the given threshold, specificity, sensitivity and area under curve (AUC) for OKS and ΔOKS anchored to patient-perceived satisfaction, function and either satisfaction or function with a cut-off of 70. True positives is the amount of patients who should be called according to the cut-off value

TKR: Cut-off value 70					
Anchor	Threshold	Called (%)	Specificity	Sensitivity	AUC
Satisfaction (*n* = 9)					
Post-operative OKS	35.5	36.8	0.71	0.78	0.78
ΔOKS	13.5	42.1	0.65	0.78	0.76
Function (*n* = 12)					
Post-operative OKS	35.5	36.8	0.76	0.83	0.85
ΔOKS	13.5	42.1	0.69	0.83	0.84
Satisfaction or function (*n* = 15)					
Post-operative OKS	35.5	36.8	0.79	0.80	0.87
ΔOKS	14.5	49.1	0.67	0.93	0.83

*n* number of patients with anchor values below cut-off

As an example, when using a cut-off value of 50 for satisfaction, a threshold of 9.5 in ΔOKS provides a sensitivity of 0.8 and a specificity of 0.83, AUC = 0.86.

All AUC values are above 0.7.

## Discussion

### Post-operative improvements

The present study found TKR patients to undergo a mean improvement in OKS of 16.5, which is consistent with findings in other studies. Judge et al. reported a mean 6–month change of 14.5 [6], whilst Beard et al. reported a change of 14.7 [7]. This demonstrates a slightly larger change in our patient group, even though mean preoperative OKS was higher in our study with 20.3 compared to 19.9 [6] and 18.5 [7], respectively.

### Thresholds

For each group, we found thresholds for two different outcome measures (post-operative OKS, change in OKS) using three different anchors (satisfaction, patient-perceived function and the combination anchor) and two different cut-offs (50 and 70). This provides additional perspectives and a better foundation for evaluating the different strengths and limitations of each threshold if they were to be used as thresholds for contacting patients. In line with previous studies [2, 6, 7], we were able to document significant correlations between the global transition items (satisfaction and patient-perceived function) and all outcome measures, justifying the use of these as anchors when establishing thresholds for the outcome measures.

Using a cut-off of 50 for each anchor, we established thresholds for change in OKS and post-operative OKS. The thresholds found in this manner were shown to have reasonable levels of sensitivity and specificity and to be consistent with results presented by Judge et al., [6] thus supporting these findings.

It may be questioned whether a cut-off of 50 is appropriate when establishing thresholds for calling patients post-operatively. Choosing a cut-off of 50 to discriminate between patients satisfied and not satisfied implies the assumption that all patients who are more than indifferent, as indicated by a score of 50, are indeed satisfied. In this respect, one may argue that patients should be more than just above “indifferent” after having undergone TKR. Similarly, patients with a function perception of 50 are not experiencing a change in function. With that in mind, we added to our analysis a higher cut-off (70) in order to detect patients who might have had a sub-optimal surgery outcome. By introducing a cut-off of 70, another set of thresholds were calculated detecting a larger proportion of patients for out-patient evaluation.

Apart from applying an extra cut-off value, we transformed the two global transition items to form one combined anchor, as it is our belief that surgery cannot be considered successful if not both satisfaction and function reach the cut-off values.

### Applicability of thresholds

Previous studies have focused on one global transition item and OKS, thus using a more simple approach to detect thresholds for satisfactory surgery outcomes. This may leave out potentially important perspectives, which this study aims to accommodate by including two different global transition items.

The purpose of previous studies has been to provide clinicians with simple and meaningful information regarding outcome after surgery and at the same time allowing a more comprehensive interpretation of OKS. Our results may be used in the same fashion; although, this has not been the main aim of our study. Instead, our approach allows us to present a variety of thresholds, using various combinations of anchors and outcome measures. In this way, we provide a large body of limits potentially useful in the clinical process of choosing patients for post-operative evaluation.

In order to make up for the sub-optimal sensitivities of the established thresholds, and thereby decrease the probability of not including all patients who might have had sub-optimal outcomes, it may be beneficial to use thresholds for both outcome measures.

A concern regarding the implementation of our thresholds as stand-alone criterions for post-operative evaluation is the considerable number of patients not in need of post-operative evaluation who are identified by the established thresholds because of specificity values below 1. This could be accommodated by an additional filter, e.g. interviewing the identified patients by phone beforehand to minimise the number of unnecessary consultations.

### Established thresholds

One of the thresholds most capable of discriminating patients into the correct group, i.e. has the highest AUC-value, is post-operative pain at rest coupled to the combination anchor. A cut-off of 50 gives a threshold of 21.5, whilst the 70-point cut-off defines a threshold of 8.5. Implementation of these thresholds would find 14 and 31.6% of all patients to be in need of out-patient evaluation, respectively. The specificity (0.90) and sensitivity (0.88) at cut-off 50 indicate that this could be a useful tool when electing patients for out-patient evaluation. At cut-off 70, specificity (0.83) and sensitivity (0.73) are lower, yielding a lower efficiency if applied in the process of electing patients for post-operative evaluation. However, this threshold may detect patients with sub-optimal improvements not identified by the threshold derived from the 50-point cut-off.

### Strengths and limitations

The sample size of 57 TKR patients is relatively small compared to that of other studies including hundreds or thousands of patients [6, 7]. As addressed previously, there is consistency between our results and those of the previous studies. This supports the assumption that our results are representative of the population.

However, as a consequence of the relatively small cohort, adjusting for confounding factors between attenders and non-attenders was not found relevant. Also, the absolute number of patients classified as eligible for evaluation is relatively low (5–15). Thus, small differences in outcome measures for these patients would have a large impact on the established thresholds. This made it impossible to yield meaningful results if patients were stratified according to age, preoperative scores, etc. This approach would be preferable, as it would have been possible to detect differentiated thresholds, e.g. based on the preoperative OKS. An alternative to stratification of patients according to preoperative scores is calculating thresholds for the percentage of potential change (PoPC) [3]. This takes into account the maximum increase possible for each patient.

As the scores range from 0 to 48, patients with a higher preoperative score have a lower potential of change than patients with a low preoperative score. As an example, if a threshold for change in OKS of 15.5 points is used as the only limit, patients with a preoperative score of 10 will not be called if their post-operative score is above 25 points. However, patients with a preoperative score of 30 points will be called even though scoring 45 points, which is close to the maximum score of 48. Furthermore, patients with a preoperative score of 34 or more will inevitably be called for evaluation, because their maximum possible improvement is 14 points. Thresholds for absolute OKS involve a similar problem, as patients with a relatively low preoperative OKS may have a big and satisfactory improvement but still not reach the threshold.

Judge et al. have shown a variance in thresholds for post-operative scores and change of OKS anchored to satisfaction when stratifying patients according to preoperative scores [6]. Further research on larger sample sizes may establish an array of thresholds based on patient groups stratified by preoperative OKS and other possible variables. This may allow the use of these thresholds as decisive for calling patients for evaluation, thus eliminating the need for the additional filter proposed previously.

Another possible limitation of the study is the follow-up period of 6 months. Previous research has shown clinical improvements in TKR patients up to 1 year after surgery, but these changes have been shown to be minor [13, 14]. Also, previous research comparable to the present study has used a 6-month follow-up to estimate clinically meaningful changes in OKS after TKR [7]. We acknowledge that a 12-month follow-up would have been preferable, but as the purpose of this study has been to develop an initial algorithm for use in a novel approach to post-operative evaluation, we believe that the 6-month follow-up is a justifiable measure in the context of this study. Thus, based on these studies, we believe that the 6-month post-operative status is a reliable indicator of long-term outcome after surgery.

In addition, it is our hope that the results of our study can be implemented as part of a post-operative battery sent to patients either by e-mail or regular mail. Therefore, a certain delay is very possible to occur from the time the forms are initially sent out until they have been answered and patients are seen in the clinic for their potential post-operative evaluation, which will then be closer to a full year after surgery.

Adding up these circumstances, we find the follow-up period of 6 months to be adequate within the aim and scope of this study.

## Conclusions

In line with the objectives of this paper, we have established a set of thresholds for the Oxford Knee Score that can be used to identify patients in need of post-operative evaluation. These clinically meaningful thresholds discriminate between patients that are satisfied with TKR surgery 6 months post-operatively and patients that are not and a similar set of thresholds differentiates between patients who have and have not experienced a gain in function after surgery.

The thresholds presented in this paper may be used when choosing limits in an at-home, web-based system comprised of questionnaires including Oxford scores, which determines whether or not to call patients for post-operative evaluation. These thresholds may require the use of an additional filter to detect patients not in need for evaluation depending on the specificity of the threshold chosen.

To establish thresholds applicable as sole determinants of which patients should be offered post-operative evaluation, we advise further research on larger sample sizes, allowing stratification of patients.

## Abbreviations

AKSS: American Knee Society Score
AUC: Area under the curve
BMI: Body mass index
CI: Confidence interval
OKS: Oxford Knee Score
PoPC: Percentage of potential change
PROMs: Patient-Reported Outcome Measures
ROC: Receiver operating characteristics
SD: Standard deviation
TKR: Total knee replacement
VAS: Visual analogue scale
